# PROTOCOL: Summarizing and Critically Evaluating the Concepts of Self‐Compassion: A Systematic Review of Conceptualizations

**DOI:** 10.1002/cl2.70054

**Published:** 2025-07-13

**Authors:** Ramona Schöne‐Hoffmann, Sarah K. Schäfer, Christina David, Dorota Reis, Manuela Benick, Franziska Perels

**Affiliations:** ^1^ Department of Educational Sciences Saarland University Saarbrücken Germany; ^2^ Department of Clinical Psychology, Psychotherapy and Psychodiagnostics Technische Universität Braunschweig Germany; ^3^ Leibniz Institute for Resilience Research Mainz Germany; ^4^ Department of Psychology Saarland University Saarbrücken Germany

## Abstract

This is a protocol for a Campbell Systematic Review. The aim of this systematic review will be to identify existing conceptualizations of general and domain‐specific self‐compassion and then integrate these into one comprehensive conceptualization. This newly developed general conceptualization will then be transferred to a domain‐specific conceptualization of academic self‐compassion.

## 1 Background

### 1.1 The Problem, Condition or Issue

Mistakes and failures are natural parts of life. We encounter them in various areas of life, including social relationships, school, work, and leisure activities. Since they are so prevalent, it is important to deal with mistakes productively or, at least, not harmfully. While it would be beneficial to approach mistakes objectively and learn from them, dealing with personal failure can present a significant challenge (Vanderheiden and Mayer 2020). Constant self‐criticism or self‐deprecation can have far‐reaching consequences. For instance, self‐criticism is linked to lower self‐efficacy after making mistakes (Stoeber et al. 2008) and reduced progress toward goals (Powers et al. 2011). A systematic review indicated a positive correlation between self‐criticism and various mental illnesses, including depression, eating disorders, and social anxiety disorders (Werner et al. 2019).

Self‐compassion, on the other hand, represents a more salutary approach to mistakes than self‐criticism. It provides a helpful solution for dealing with one's shortcomings and can be understood as an adaptive self‐reaction following negative events (Neff 2003; Neff et al. 2005). Neff (2003) was the first to define the construct in psychological research. She divided self‐compassion into three components: self‐kindness, mindfulness, and common humanity. *Self‐kindness* encompasses a benevolent and understanding disposition toward one's self in lieu of negative and self‐critical reactions. *Common humanity* describes one's understanding of suffering as an integral part of human existence and a phenomenon shared across mankind, thus recognizing that one is not isolated in one's suffering. Finally, according to Neff (2003), *mindfulness* is the act of perceiving stressful thoughts with mindful awareness, not allowing oneself to become wholly absorbed by them. A more developed version of Neff's (2003) conceptualization encompasses the negative antipoles for each component – self‐judgment versus self‐kindness, isolation versus common humanity, and overidentification versus mindfulness (Neff 2016). The extant research on self‐compassion is largely based on this understanding of the construct (Muris and Otgaar 2020). To offer an initial understanding of the conceptual diversity of the construct, two additional conceptualizations are presented below. The first is a subsequent elaboration of Neff's conceptualization (Dodson and Heng 2021), while the second exemplifies a conceptual integration of compassion and self‐compassion (Strauss et al. 2016).

Dodson and Heng's (2021) recent conceptualization described self‐compassionate responses as a dynamic process that encompasses three subprocesses: noticing, feeling, and acting. In line with the mindfulness component of Neff's (2003) conceptualization, *noticing* is defined as acknowledging one's suffering without becoming overwhelmed. The process of *feeling* describes the act of being empathetic toward oneself and acknowledging one's suffering as valid. Lastly, *acting* encompasses taking actions to overcome pain and regain well‐being.

Another conceptualization of self‐compassion was presented by Strauss et al. (2016). These authors described self‐compassion and compassion for others as a process comprising cognitive, affective, and behavioral components, divided into five aspects: (1) the ability to recognize one's own suffering, (2) an understanding of suffering as a universal human experience, (3) empathy, (4) the capacity to tolerate unpleasant feelings to ensure openness and acceptance of emotional situations, and (5) the motivation to act in a way that reduces suffering.

A further range of conceptualizations of self‐compassion exists (e.g., Gilbert et al. 2017; Keller and Huppert 2021). These conceptualizations partially overlap but are still inherently different. Some researchers have criticized Neff's (2016) conceptualization for including negative components within a “positive” and salutogenic construct like self‐compassion (e.g., Büchner et al. 2025; Muris and Otgaar 2020). A recent study by Büchner et al. (2025) undertook a more detailed examination of this issue and concluded that it might be more appropriate to restrict the conceptualization of self‐compassion to its positive components and utilize the negative counterparts that Neff (2016) defined to measure the effectiveness of self‐compassion interventions.

Some authors have viewed self‐compassion as equivalent to compassion for others (e.g., Gilbert et al. 2017; Strauss et al. 2016), whereas other researchers have derived the construct of self‐compassion from general compassion but narrowed it to a qualitatively distinct process (e.g., Neff 2003). Nevertheless, the constructs of compassion for others and self‐compassion share a common origin, the Buddhist worldview. Here, compassion for others as well as for oneself is regarded as openness to suffering and a commitment to relieve that suffering (Lama 2002). A nonjudgmental attitude toward suffering and the ability to tolerate suffering play a key role in Buddhism. This common origin of both constructs suggests a common conceptualization of compassion for others and self‐compassion in psychological research. However, research on the relationship between scales measuring compassion for others and self‐compassion has produced ambiguous findings (e.g., Gu et al. 2019; López et al. 2018). This ambiguity may suggest potential differences in the underlying components or processes when individuals exhibit compassionate behavior toward others compared to themselves (Halamová et al. 2022; Sinclair et al. 2017).

Cultural differences may also influence the conceptualization of self‐compassion. A meta‐analysis by Chio et al. (2021) compared the associations between the components of self‐compassion as defined by Neff (2003) in 27 different countries. The findings indicated that individuals from dialectic cultures (societies that accept that there is no one absolute truth and that opposing perspectives can coexist, e.g., East Asian societies) appeared to experience self‐compassion somewhat differently than individuals from analytic cultures (societies that emphasize logic, consistency, and categorization, e.g., Western cultures). Neff (2023) further postulates that cultures with closer affinities to Buddhism may have a more positive perspective on self‐compassion and its practice. Furthermore, researchers acknowledge the potential influence of culture on the perception of self‐compassion and address the lack of data in this domain as a shortcoming of the field (e.g., Gu et al. 2019; Steindl et al. 2021).

In summary, no comprehensive conceptualization of self‐compassion exists to date, and researchers remain divided about what self‐compassion really is (Halamová et al. 2022; Pastore et al. 2023). Therefore, the main topic of this systematic review will be the conceptual ambiguity surrounding the various conceptualizations of self‐compassion. Hence, we will present the conceptualizations that currently exist in the literature and offer an integrative conceptualization of general self‐compassion.

Concerning the importance of self‐compassion for different areas of life, there are associations with various constructs from the domain of health and well‐being. For instance, a meta‐analysis based on Neff's (2003) conceptualization, showed negative correlations between self‐compassion and anxiety, depression, and stress in adolescents (Marsh et al. 2018). Similarly, a recent systematic review found that self‐compassion interventions are effective in alleviating anxiety and depression in adolescents (Egan et al. 2022). The interventions examined in this review referred to Neff's (2003) and Gilbert et al.'s (2017) conceptualizations of self‐compassion. The effectiveness of self‐compassion interventions is further supported by a recent meta‐analysis, which demonstrated small to medium positive effects of self‐compassion interventions on depression, stress and anxiety in various populations (Han and Kim 2023). Associations with better health and well‐being have also been found in older populations: In a study of adults aged between 63 and 97 years with health impairments, self‐compassion as conceptualized by Neff (2003) was found to have a positive association with subjective well‐being and the use of aids to improve quality of life (Allen et al. 2012).

Moreover, self‐compassion can have a positive effect on one's working environment. The findings of a longitudinal study indicated that self‐compassion serves as a protective factor against burnout by buffering workplace exhaustion (Schabram and Heng 2022). Again, the authors referred to Neff's conceptualization. Furthermore, a study with Japanese workers demonstrated a negative relationship between self‐compassion as conceptualized by Neff (2003) and mental health problems, showing an additional negative link with amotivation (Kotera et al. 2022). These findings suggest that self‐compassion might be able to help workers improve their mental health and thus reduce amotivation.

A scoping review found that self‐compassion as conceptualized by Neff (2003) is associated with improved well‐being in social relationships (Lathren et al. 2021). That is, individuals who exhibit greater self‐compassion tend to adopt a more constructive approach to interpersonal conflicts.

In addition to these beneficial links, self‐compassion can be advantageous, especially in educational settings, where errors and setbacks hold significant value. Mistakes can help students to use existing competencies more efficiently, or stimulate the development of new competencies (Käfer 2022). However, these beneficial effects only occur when students are not discouraged or overwhelmed by negative emotions. Therefore, it is crucial to cultivate adaptive self‐reactions to mistakes and failure. Self‐compassion can thus help one to disengage from such negative emotions (Yip and Tong M. W. 2020).

Moreover, research has indicated that self‐compassion is positively associated with several favorable factors for learning and education, including goal orientation (Babenko and Oswald 2018) and self‐efficacy (Liao et al. 2021). Self‐compassion can also improve students' well‐being (Seekis et al. 2023) and is associated with learning outcomes (Egan et al. 2021). Furthermore, training student teachers in self‐compassion might be a promising way to implement self‐compassion at all levels of education, especially since student teachers are important role models and can have multiplying effects (Jennings 2014). In accordance with the predominance of Neff's conceptualization in research, the aforementioned four studies similarly referenced her conceptualization. However, Liao et al. (2021) were the only ones to acknowledge the existence of additional conceptualization proposals.

To summarize, self‐compassion plays an important role in central aspects of everyday life. While much research has considered self‐compassion a trait that is relevant for various life domains, more recently, researchers have been focusing on domain‐specific components of self‐compassion (Neff 2023; Pastore et al. 2023). Following these approaches, self‐compassion may not only be a way of treating oneself in general; it may also manifest in different ways depending on the context. Here, Zuroff et al. (2021) modified the items of the well‐established short form of the general Self‐Compassion Scale (SCS‐SF; Raes et al. 2011) to measure domain‐specific self‐compassion in eight domains (i.e., relationships with family and friends, romantic relationships, relationships at school or work, handling one's finances, academic or professional performance, physical appearance, and health). Their findings indicate that levels of self‐compassion are not consistent across domains. Consequently, examining general self‐compassion alone may not be sufficient, particularly when conclusions need to be drawn for specific domains (e.g., how self‐compassionate students behave in academic contexts). This finding is supported by the results of another study that used a domain‐specific modification of the SCS‐SF to measure self‐compassion in interpersonal relationships (also termed “social self‐compassion”; Rose and Kocovski 2020) in comparison to general self‐compassion. The study found that the domain‐specific version of the scale can explain unique variance beyond general self‐compassion in specific social outcome variables. These findings suggest that further research on domain‐specific self‐compassion is needed.

In line with this idea, some authors have started to conceptualize domain‐specific self‐compassion constructs. For instance, Altman et al. (2017, 441) defined the construct of *body compassion* as “the regarding of one's own body, in appearance, competence and health, with mindfulness, kindness and awareness of common humanity” (for a similar approach, see Beadle et al. 2021). Other domain‐specific concepts of self‐compassion relate to work. For example, *leader role self‐compassion* reflects a leader's attitude, which involves treating themselves with kindness and patience while recognizing the challenges and difficulties that come with leadership (Lanaj et al. 2022). These domain‐specific conceptualizations have primarily been based on Neff's (2003) general concept of self‐compassion. For the academic field, though, no domain‐specific conceptualization has been proposed yet. However, such a conceptualization could provide further insights into self‐compassion in educational settings. Through the development of a domain‐specific conceptualization, the relationships to other academic constructs could be explored in more detail.

Therefore, in this systematic literature review, we will focus on conceptualizations of general and domain‐specific self‐compassion to provide a comprehensive overview and critical examination of existing conceptualizations, using the findings to develop an integrative conceptualization of general self‐compassion that can be transferred to the academic context. The authors of previous conceptualizations have made a significant and invaluable contribution to the field of research on this construct. The integrative conceptualization that we aim to provide should reflect the interest of future integrative research.

Notably, what is meant by *conceptualization* is not agreed upon in the literature, but we understand it to be an umbrella term that encompasses definitions, models, and theories, as well as the process of establishing concepts for abstract processes (APA Dictionary of Psychology 2018).

### 1.2 Why It Is Important to Do This Review

Numerous attempts have been made to conceptualize the construct of self‐compassion (e.g., Dodson and Heng 2021; Gilbert et al. 2017; Neff 2003; Strauss et al. 2016), and several authors have commented on the problems associated with previous, only partially overlapping conceptualizations of self‐compassion, calling for a revision of the construct (e.g., Büchner et al. 2025; Ferrari et al. 2022). A comprehensive conceptualization that integrates relevant theoretical and empirical components is still needed (Pastore et al. 2023). This gap in research makes it challenging to explore self‐compassion in depth and develop appropriate methods for its measurement and interventions for its promotion. Furthermore, the conduction of meta‐studies can be impeded: Systematic reviews and meta‐analyses might include research on heterogeneous concepts, thereby placing themselves at risk of comparing two vastly different concepts. Alternatively, researchers may impose limitations to their work by focusing on only one of the many conceptualizations, thereby inherently excluding other studies that explore the same construct.

With our systematic review, we aim to provide the conceptual clarity that is currently lacking. Our first objective is to synthesize the current scientific conceptualizations of general self‐compassion as well as to summarize domain‐specific conceptualizations of self‐compassion. Second, to establish a consensus among the conceptualizations outlined in the literature review, an integrative conceptualization of general self‐compassion will be proposed. Third, we will apply this conceptualization to the context of education to develop a domain‐specific conceptualization of self‐compassion (i.e., *academic self‐compassion*).

Providing an exhaustive overview of the research on conceptualizing self‐compassion at present and developing a theoretically integrative, coherent, and comprehensive conceptualization of self‐compassion may have the following implications for the field:

1. Implications for defining self‐compassion: By contrasting, analyzing, and integrating different conceptualizations of self‐compassion, we can promote conceptual clarity within the field. Additionally, the heterogeneous and partly contradictory findings about the nature of the construct from previous research may become explainable.
2. Implications for further research: A comprehensive conceptualization of self‐compassion can offer a foundation for improved comparability in research if future studies base their measurement of self‐compassion on the same conceptualization of the construct. This may lead to replicable findings and a more solid evidence base for self‐compassion and its associations with other constructs (e.g., compassion towards others, academic achievement, procrastination).
3. Implications for practical application: A comprehensive conceptualization is also useful for interventions by offering a more precise delineation of the components that are integral to the construct and which are not. Given the growing interest in interventions that at least partially incorporate self‐compassion, a comprehensive conceptualization of self‐compassion will facilitate future research endeavors aimed at elucidating the underlying mechanisms of change.
  Although domain‐specific versions of self‐compassion have been defined for other areas of life (e.g., body compassion, work self‐compassion; Altman et al. 2017; Jennings et al. 2022), there is currently no such construct for the academic context. Transferring a general conceptualization of self‐compassion into a domain‐specific conceptualization of academic self‐compassion may have specific implications for the educational sector.
4. A domain‐specific conceptualization of academic self‐compassion can be used to develop an appropriate instrument to measure self‐compassion in academic contexts and explore the relationships between self‐compassion and other constructs that have been shown to be important in the educational context (e.g., motivation, self‐efficacy, goal setting).

### 1.3 How This Review Might Inform or Supplement What Is Already Known in This Area

There have been several attempts to summarize conceptual work on self‐compassion. A systematic search for previous reviews was conducted using the following databases and search terms:

- PROSPERO: “Self‐compassion”.
- PsychArchives: “Self‐compassion”.
- Web of Science: “Self‐compassion,” document type “review”.
- Open Dissertations, PsycArticles, PsycInfo, PSYNDEX, ERIC (via EBSCOhost): “Self‐compassion AND review”.
- Campbell and Cochrane Reviews: “Self‐compassion”.

This search resulted in the identification of 14 relevant reviews. However, these reviews have several limitations, which will be addressed in our proposed review:

1. Some reviews have not used a systematic approach for their literature search, data extraction, synthesis, and/or reporting (Barnard and Curry 2011; Muris and Otgaar 2020; Neff 2023).
2. Some reviews have focused on reviewing assessments of self‐compassion rather than underlying conceptualizations (Strauss et al. 2016).
3. Some reviews have only focused on specific settings or populations, such as self‐compassion among healthcare providers (Sinclair et al. 2017) or in organizational contexts (Dodson and Heng 2021).
4. Some reviews have been limited in their selection of databases used for the literature search, while some have not included sources other than empirical or theoretical research (Muris and Otgaar 2020; Sinclair et al. 2017; Swami et al. 2021).
5. The rapidly growing number of studies on self‐compassion has made an exhaustive review of the literature on the conceptualization of self‐compassion challenging. Hence, systematic searches for relevant keywords often lead to an extremely large number of hits per database (Strauss et al. 2016).

To the best of our knowledge, and based on a search of PROSPERO and the Open Science Framework, no systematic literature review is yet published that provides a comprehensive overview of existing conceptualizations of general and domain‐specific self‐compassion. Furthermore, a comprehensive conceptualization of general self‐compassion is still needed (Pastore et al. 2023). The limitations of former reviews on the topic will be addressed using the following measures:

1. We will adhere to a systematic procedure for the identification of eligible studies following standards for conducting high‐quality systematic reviews (i.e., Guide to information retrieval for Campbell systematic reviews; MacDonald et al. 2024; MECCIR conduct and reporting standards; Wilson et al. 2024; PRISMA standards for the reporting of systematic reviews; Page et al. 2021; SRQR Standards for reporting qualitative research; O'Brien et al. 2014).
2. We will search for a wide range of databases to enable a comprehensive coverage of works on existing conceptualizations.
3. We will use advanced AI such as Rayyan (Ouzzani et al. 2016) to synthesize evidence and overcome the challenge of large numbers of hits per database.
4. We will search for conceptualizations of general and domain‐specific self‐compassion. The instruments for the assessment of self‐compassion will not be the subject of this review, thus allowing us to clearly separate conceptualization from measurement problems.
5. Eligible conceptualizations will be systematically recorded and analyzed for included components and their interaction and theoretical foundations.
6. Key elements will be identified and integrated into a theoretically sound and comprehensive conceptualization of general self‐compassion.
7. This comprehensive conceptualization will then be applied to the field of education to develop a domain‐specific conceptualization of academic self‐compassion.

## 2 Objectives

The aim of this systematic review will be to identify existing conceptualizations of general and domain‐specific self‐compassion and then integrate these into one comprehensive conceptualization. This newly developed general conceptualization will then be transferred to a domain‐specific conceptualization of academic self‐compassion.

First, we will conduct a comprehensive search for conceptualizations of general and domain‐specific self‐compassion. The research questions related to this synthesis will be as follows:

a. Which conceptualizations of general self‐compassion exist to date?
b. Which domain‐specific conceptualizations of self‐compassion exist to date?
  Second, the conceptualizations of general self‐compassion identified will be analyzed in terms of their core elements and theoretical foundations. Subsequently, the conceptualizations will be compared. This analysis will involve identifying recurring core components, as well as components that require critical consideration. Based on these findings, a theoretically sound, comprehensive, and integrative conceptualization of general self‐compassion will be developed. The following research question will guide this step of the review project.
c. Which key components of the conceptualizations of general self‐compassion can be identified and incorporated in an integrative and comprehensive conceptualization?
  The third part of the systematic literature review will address the transfer of the newly developed conceptualization of general self‐compassion to the academic field. This process will be guided by the following research question.
d. What key components are incorporated within the domain‐specific conceptualization of academic self‐compassion, as per previous research on domain‐specific self‐compassion and the newly developed comprehensive conceptualization of general self‐compassion?

## 3 Methods

### 3.1 Criteria for Considering Studies for This Review

The inclusion and exclusion criteria for this systematic literature review are described in detail below. For a brief overview of the criteria, please refer to Table 3.

#### 3.1.1 Types of Studies

To ensure a comprehensive documentation of current conceptualizations of self‐compassion, we will include both theoretical and empirical research from the fields of psychology and educational science with any study design. The following study designs will thus be acceptable:

- Empirical research following cross‐sectional and longitudinal designs using quantitative and qualitative methods.
- Theoretical research (e.g., narrative reviews, position papers).
- Systematic and narrative reviews and meta‐analysis.
- Other document types, including commentaries, editorials, letters to the editor, and book chapters.
- Gray literature, including theses, doctoral dissertations, study protocols, research reports, preprints, and other unpublished research.

This decision was made based on initial searches (nonsystematic, conducted in preparation for this study to ensure a comprehensive understanding of the issue), that showed that empirical as well as theoretical studies encompass conceptualizations of (domain‐specific) self‐compassion.

Since psychological research on self‐compassion started with the work of Neff (2003), publication dates will be limited to 2003 to the present. Earlier texts on self‐compassion originated in philosophy and Buddhism and will thus not be included in this review.

Due to the language proficiency of the authors, articles with full texts or relevant text passages (i.e., those containing a conceptualization of general or domain‐specific self‐compassion) available in English or German will be considered for this systematic literature review. Nevertheless, to consider the possibility of language‐related limitations, excluded articles published in other languages yet still considered eligible for a full‐text analysis at the title or abstract level will be documented and reported.

Regarding the first objective of this systematic review—to identify and integrate current conceptualizations of general and domain‐specific self‐compassion—studies with one of the following aspects in their title or abstract will be considered for full‐text screening:

- Studies that present a new conceptualization, development, or adaptation of a previous conceptualization of general or domain‐specific self‐compassion that is either empirically tested or based on theoretical considerations (i.e., conceptualizations for which no model checks based on empirical data are available or for which no instrument has yet been developed).
- Studies that discuss the conceptualization of general or domain‐specific self‐compassion.
- Studies that point to the conceptual ambiguity of self‐compassion.

Only studies that present a new or refined conceptualization of general or domain‐specific self‐compassion will be eligible for inclusion. Studies that do not present their own conceptualization or do not develop an existing conceptualization, as well as those that do not clearly identify the origin of the conceptualizations presented, will be excluded. However, the former two types of studies will be used to identify additional relevant literature sources. Conceptualizations consisting of vernacular definitions (i.e., based on surveys of specific populations) will be excluded.

For example, the following two studies will be eligible for inclusion and provide insight into the range of methodologies employed in relevant studies:

1. Strauss et al. (2016): A review presenting conceptualizations of compassion and self‐compassion and systematically comparing them based on postulated components. A new conceptualization of self‐ and other‐compassion was developed.
2. Lanaj et al. (2022): An experimental study developing and empirical testing a domain‐specific conceptualization (leader role self‐compassion).

#### 3.1.2 Types of Participants

We will include all types of populations, irrespective of their age, gender, ethnicity, nationality, and mental or physical health status. The rationale for including all populations is that we will be focusing on potential domain‐specific conceptualizations of self‐compassion, and these domain‐specific conceptualizations are more likely to be embedded in studies examining specific populations.

#### 3.1.3 Phenomenon of Interest

This review will identify the components that constitute self‐compassion, aiming to develop an understanding of their interactions to provide an integrative description of what self‐compassion entails. Therefore, we will focus on conceptualizations of self‐compassion. We will exclude theories that focus solely on the development of self‐compassion in humankind or within a person, as well as models that represent the relationship between self‐compassion and other constructs without providing a conceptualization of the construct itself.

To fulfill the objectives (a) and (b), we will document all available conceptualizations of general and domain‐specific self‐compassion. We will determine whether a new conceptualization is newly developed or built on an existing conceptual framework. Individual components of the conceptualizations will then be determined, in addition to statements and assumptions about the interactions among the components. The domains of domain‐specific conceptualizations of self‐compassion will be identified. Furthermore, to what extent they overlap with and/or differ from the conceptualizations of general self‐compassion will be explained. The aim here will be to examine whether a domain‐specific conceptualization represents a transfer of general self‐compassion to a specific domain or whether it is based on domain‐specific theoretical assumptions and has been enriched with domain‐specific components.

In this systematic literature review, we will not assess the frequency of the use of specific conceptualizations in research. Consequently, studies that only mention existing conceptualizations of general or domain‐specific self‐compassion without modifying or expanding them will be excluded.

Conceptualizations derived from surveys of specific populations and based on interview content (i.e., vernacular definitions) will also be excluded.

#### 3.1.4 Context

Apart from the general construct of compassion toward others, research on self‐compassion has also included other related constructs such as *Compassion satisfaction* (Mooney et al. 2017; Stamm 2012), *Compassion fatigue* (Figley and Ludick 2017; Stamm 2012) and *Fear of (self‐)compassion* (Gilbert et al. 2011). Neither of these will be included in this systematic review as we focus on the conceptualization of self‐compassion only.

Some conceptualizations of self‐compassion include the component of mindfulness (e.g., Neff 2003). However, mindfulness is a separate psychological construct that has been the subject of extensive research. Conceptualizations of mindfulness will thus not be included in this systematic review; it will only be viewed as a potential component of self‐compassion.

Another significant area of research in the field of self‐compassion is the operationalization and assessment of self‐compassion. Since Neff's (2003) development of the first empirical instrument to measure general self‐compassion, various questionnaires have been developed to measure general and domain‐specific self‐compassion. Regarding Neff's (2003) original scale, there have been repeated discussions about its appropriateness. Criticisms have mainly focused on the inclusion of the negative counterparts of the three self‐compassion components (self‐kindness vs. self‐criticism, common humanity vs. isolation, mindfulness vs. overidentification; Neff 2016). Concerning the numerous discussions about the factor structure of SCS, Ferrari et al. (2022) argued that it is more likely to be a conceptualization problem than an operationalization problem. Therefore, in this systematic review, we will focus on the conceptualization of self‐compassion rather than its operationalization, with our results likely inspiring future discussions about appropriate operationalization.

### 3.2 Search Methods for Identification of Studies

#### 3.2.1 Electronic Searches

Our search strategy was developed in accordance with the recommendations for conducting narrative reviews, meta‐analyses, and meta‐syntheses presented by Siddaway et al. (2019) as well as the guide for information retrieval for Campbell systematic reviews by MacDonald et al. (2024), adapted to the objectives of this systematic review through discussions within the review team. As MacDonald et al. (2024) have suggested, in the event that an Information Specialist (IS) is not included in the research team, it is advisable to consult with them before finalizing the search strategy. Given that the review process of Campbell's Systematic Reviews incorporates feedback from an IS, this limitation can be addressed through the conscientious implementation of the proposed recommendations within the peer‐review process. Furthermore, the development of the search strategy was guided by conducting and reporting guidelines, including the Guide to Information Retrieval for Campbell Systematic Reviews (MacDonald et al. 2024), the MECCIR Conduct and Reporting Standards (Wilson et al. 2024), the PRISMA Standards for Reporting Systematic Reviews (Page et al. 2021), and the Standards for Reporting Qualitative Research (SRQR; O'Brien et al. 2014). Significant phases in the design of the search process, such as the selection of databases and gray literature searches, the construction of the search term, and the procedure for securing data, were largely led by a team member with a high level of expertise in the field of systematic reviews (S.K.S.).

The following electronic databases will be searched for studies published between 2003 and the present: Web of Science (Core Collection), PubPsych (PSYNDEX, NARICS, ISOC‐Psicologia, Pascal, PsychOpen), APA PsycArticles (via EBSCOhost), APA PsycInfo (via EBSCOhost), ERIC (via EBSCOhost), OpenDissertations (via EBSCOhost), PubMed, and Scopus. Apart from these databases, the publishing platform SAGE Journals will be used. It provides access to high‐impact journals that may not be fully indexed in other databases. In contrast to databases that primarily focus on empirical research, SAGE includes a significant number of qualitative, theoretical, and conceptual studies. This makes it a valuable addition to our systematic review. These databases were selected since they include a wide range of relevant journals from the fields of psychology and educational science, in addition to gray literature such as dissertations, preprints, conference proceedings, and study protocols (see Table 1 for an overview of all sources of electronic search).

**Table 1:** List of databases for electronic searches.

Databases hosted by Web of Science Web of Science Core Collection Databases hosted by PubPsych: ∘ PSYNDEX ∘ NARCIS ∘ ISOC‐Psicologia ∘ Pascal ∘ PsychOpen Databases hosted by EBSCOhost: ∘ APA PsycArticles ∘ APA PsycInfoERIC ∘ OpenDissertations Databases hosted by PubMed: ∘ Medline SAGE Journals Scopus

The search strategy will comprise two clusters of search terms related to:

1. Self‐compassion.
2. The conceptualization of psychological constructs.

The keywords were collected based on terms used in studies already identified as relevant and using a dictionary to identify different spellings and synonyms. Care was taken to include different spellings from different regions and to work with truncations and wildcards according to the possibilities of the databases searched. Terms within one cluster will be linked using the Boolean operator OR, while two clusters will be combined using the Boolean operator AND. For an extensive overview of the search terms, see Table 2.

**Table 2:** Search terms for electronic searches.

Cluster (1)	Cluster (2)
self‐compassion “self compassion” selfcompassion self*compassion self?compassion	defin* concept* theor* model* expla* characteriz* clarif* determin* formali* terminolog* elucidat* outlin* construct* descri* understand* breakdown “breaking down” “broken down” account* establish* analyz* dissect* approach* propos* discuss* consider* idea* term*

The search strategies for each database are presented in Appendix S1. The search operators permitted by the database were identified, and the search term was adapted accordingly. Furthermore, the search will be conducted in accordance with the availability of the “area search” function. Ideally, the title, abstract, and keywords of the individual entries will be searched. If possible, the publication period will be limited to 2003 to the present. As postulated by MacDonald et al. (2024), the development of the search strategy entailed the identification of various “benchmark studies” (i.e., Neff 2003; Strauss et al. 2016; Dodson and Heng 2021) among the results to verify the fit of the search strategy.

For full reproducibility, the following information will be recorded:

- Date of the search (dd/mm/yyyy).
- Title of the database searched.
- Search term used.
- Number of search results obtained.

#### 3.2.2 Searching Other Resources

Further search strategies will be implemented to reduce the impact of biases and guarantee that all relevant articles are identified. This will involve the following procedures:

- Manual searches of the reference lists of reviews identified in the preliminary literature search outlined in section “How this review might inform or supplement what is already known in this area”.
- Manual searches of the reference lists of all eligible studies, as well as those of articles excluded during the full‐text screening stage, for not providing new conceptualizations.
- Manual searches of all publications of authors who have created relevant conceptualizations of general or domain‐specific self‐compassion to identify possible developments of these conceptualizations.
- Manual searches of all studies citing those studies eligible for inclusion using the “cited by” function of Web of Science.

For full transparency during additional searches, the following information will be documented:

- Date of the search (dd/mm/yyyy).
- Sources searched (e.g., publication lists, reference lists).
- Name of the researcher conducting the search.
- Number of potentially relevant and eligible studies identified during the additional search.

### 3.3 Data Collection and Analysis

#### 3.3.1 Description of Methods Used in Primary Research

To answer the research questions of this systematic review, relevant text passages with conceptualizations of general or domain‐specific self‐compassion will be extracted from primary studies. The existing conceptualizations will be analyzed using an inductive category development strategy to compare their structure and components, the assumed interactions among these components, and their theoretical foundations. Consequently, any research that presents a new and unique conceptualization of the construct of general or domain‐specific self‐compassion as text or a figure, in which the individual components of the construct are identifiable, will be of particular interest.

Our initial literature search (nonsystematic, conducted in preparation for this study to ensure a comprehensive understanding of the issue) suggested that conceptualizations of general self‐compassion are often found in qualitative or theoretical studies, whereas domain‐specific conceptualizations are more frequently found in quantitative studies (e.g., Jennings et al. 2022; Lanaj et al. 2022; Altman et al. 2017). For a comprehensive understanding of the type of research and the structure and methods employed in the studies of interest, two studies on general and domain‐specific self‐compassion are presented below. These two studies contain relevant conceptualizations, which are also presented below:

- “Self‐Compassion: An Alternative Conceptualization of a Healthy Attitude Toward Oneself” by Kristin Neff (2003).

Neff presented a theoretical paper in which she discussed the origins of the construct of compassion, upon which she then based her own conceptualization of self‐compassion:

Self‐compassion, therefore, involves being touched by and open to one's own suffering, not avoiding or disconnecting from it, generating the desire to alleviate one's suffering and to heal oneself with kindness. Self‐compassion also involves offering nonjudgmental understanding to one's pain, inadequacies and failures, so that one's experience is seen as part of the larger human experience.

(p. 87)

Neff subsequently addressed potential concerns about her newly defined construct and compared self‐compassion with other constructs from different domains (e.g., well‐being or educational settings).

- “The body compassion scale: Development and initial validation” by Altman et al. (2017).

The empirical study aimed at validating a newly developed instrument for the construct of *body self‐compassion*. The authors defined *body self‐compassion* as follows:

[…] the regarding of one's own body, in appearance, competence and health, with mindfulness, kindness and awareness of common humanity.

(p. 441)

Given the significant discrepancies in methodologies and the overall quality of the primary studies, it is likely that this systematic review will have certain limitations. One of these limitations might be the significant variations in the level of detail of the conceptualizations. Preceding (nonsystematic) literature searches revealed that conceptualizations range between having great detail (e.g., “self‐compassion in working contexts” in Dodson and Heng 2021) to having limited theoretical foundations for the assumptions made, particularly for domain‐specific self‐compassion (e.g., “leader self‐compassion” in Lanaj et al. 2022).

#### 3.3.2 Selection of Studies

In accordance with MacDonald et al. (2024), the study selection process will involve saving the search results from each database, removing duplicates, screening titles and abstracts, collecting full‐texts, and screening full texts. The entire screening process will be conducted using Zotero and the web‐based software Rayyan (Ouzzani et al. 2016). Rayyan offers integrated AI tools that will be used to facilitate and accelerate the screening process. Many studies have been conducted on the efficacy and reliability of Rayyan (i.e., Reis et al. 2023; Valizadeh et al. 2022), demonstrating that Rayyan exhibits especially good performance in screening compared to other freely available software, with particularly good sensitivity. Given the complexity of the subject matter addressed in this review and its unsystematic integration into primary sources, it is essential to prioritize the sensitivity of the screening process. Additionally, the program allows for highlighting keywords in study abstracts. These highlights are defined in advance and differ in color depending on whether they match the inclusion or exclusion conditions. This function will be used to facilitate the abstract and title screening process. Furthermore, the program allows for assessing the probability of the inclusion or exclusion of studies. This function increases the efficiency of the screening process and is particularly useful when there are large numbers of records. The probability rating is based on the screener's initial rating of a smaller number of studies, following which the program provides recommendations aligned with the original evaluation criterion, thus supporting the consistency of the rating. It is crucial to emphasize that no records are omitted from the screening process, thereby ensuring that the ultimate decision regarding inclusion always resides with a researcher and never with the AI system.

To ensure the standardized assessment of individual records, screening guidelines were developed (see Appendix S2). This document incorporates inclusion and exclusion criteria with further instructions for the labeling of records with reasons for exclusion at the full‐text level or how to handle missing abstracts and full texts. Notably, some of the defined inclusion and exclusion conditions only become relevant at the full‐text level (e.g., the identification of a conceptualization as a theory). The corresponding conditions are clearly noted as such in the screening guidelines. This document was discussed in detail with all reviewers involved, as suggested by MacDonald et al. (2024). In addition, the inclusion criteria were tested on various studies that were certain to be included in our review (i.e., Neff 2003; Strauss et al. 2016; Dodson and Heng 2021) to ensure that they would pass the selected criteria.

1. Deduplication: Following the saving of search results from databases in the form of .ris or .bib files, files will be imported into Zotero, where they will be merged and screened for duplicates using the built‐in Zotero feature. The remaining search results will then be exported from Zotero as a .ris file and imported into Rayyan. Once imported into Rayyan, the studies will again be checked for duplicates using the built‐in Rayyan algorithm. The identification and removal of duplicates will be carried out by the main author, R.S.‐H. As stated by MacDonald et al. (2024), this step is not only important because of overlapping results from different databases, but also because some studies are reported in multiple publications, which might lead to redundant search results.
2. Title and Abstract Screening to identify clearly irrelevant records: A total of four scientists will be involved in the screening process: two of the review authors (R.S.‐H. and C.D.) and two research assistants. This should ensure appropriate expertise and, simultaneously, that the expected high volume of records will be screened in a reasonable amount of time. All screeners involved are fluent in English and have received training in methods of psychological research. The review team members will independently screen the titles and abstracts of identified records to assess their eligibility for full‐text screening. Irrelevant records will be excluded immediately. A trial screening of a random sample of search results will be conducted. This trial run will be considered successful if an agreement of at least 90% in decisions is achieved. Disagreements will be discussed after the trial screening, and final decisions will be documented. After successfully conducting the trial screening, screeners will then start the title or abstract screening in Rayyan. Abstracts provided in languages other than English or German will be translated using an AI‐based translation tool (DeepL). Furthermore, “border cases” will be discussed regularly, while interrater reliability will be calculated and reported via Cohen's kappa.
3. Full‐Text Screening: Two members of the review team will again independently check the eligibility of relevant articles at the full‐text level. A local electronic copy of all articles of this step will be saved, as access might cease (MacDonald et al. 2024). If studies are excluded at this final stage, the reasons for this decision will be documented for each study (see screening guidelines page 2 for a list of labels for exclusion reasons). Studies included as relevant based on their abstract during the title or abstract screening stage but whose full texts are only available in a language other than German or English will be documented to derive information about potential language bias. These studies will be excluded from further analysis, though, as we will not be able to guarantee the accuracy of AI‐based translations. Since the review question specifically focuses on the detailed analysis of texts, reliance on translations of full texts will be a limitation of this review. In the event that full texts can't be obtained despite exhaustive efforts, such instances are duly documented in the PRISMA flow chart. Again, interrater reliability will be calculated and reported via Cohen's kappa.

The inclusion and exclusion criteria are presented in Table 3. The screening process will be reported as a PRISMA flow chart (Page et al. 2021).

**Table 3:** Inclusion and exclusion criteria.

*Inclusion criteria* – Records including any population/sample regardless of age, gender, other socio‐demographic characteristics, mental and physical health status – Record was published in 2003 or later – Abstract is available in any language; record is available in English or German – Record can be classified as empirical research (including cross‐sectional or longitudinal designs using quantitative and qualitative methods, experimental studies including randomized controlled trials (RCTs) and cluster‐randomized trials (cRCTs), pilot studies, correlational studies, descriptive studies), theoretical research (including narrative reviews, position papers, discussions), systematic reviews and/or meta‐analysis, theses or doctoral dissertations, research reports, preprints, preregistrations or study protocols, commentaries, conference papers or posters, letters to the editor, editorial or book chapters – Record presents a conceptualization of general or domain‐specific self‐compassion, which is either empirically tested OR merely based on theoretical considerations – Record describes, introduces, or discusses the construct of general or domain‐specific self‐compassion – Record discusses the conceptual ambiguity of self‐compassion
*Exclusion criteria* (can only be assessed at full‐text level) – Record only reproduces an existing conceptualization of general or domain‐specific self‐compassion without further developing it or providing a new original conceptualization of self‐compassion – Record only reports on nonscientific/vernacular conceptualizations of self‐compassion (based on input from the general population or specific populations) – Record only presents a theory on the development of self‐compassion in mankind or within a single person – Record only presents models that represent the relationship between general or domain‐specific self‐compassion and other constructs (e.g., moderation or mediation models, psychological network models)

#### 3.3.3 Data Extraction and Management

Two reviewers (R.S.‐H. and C.D.) will independently conduct the data extraction process. To ensure comprehensible and systematic data extraction, a standardized coding scheme was developed (see Appendix S3). Any discrepancies will be resolved through discussion, and final decisions will be reported. The following information about the included studies will be extracted:

- Full citation information (including information about the authors, year of publication, journal, or publisher).
- Document type (empirical or theoretical research, meta‐analysis, gray literature, other).
- Language.
- Country of origin of record and author(s).
- Profession of author(s).
- Focus of the document (self‐compassion or other topic).
- Area of conceptualization (general or domain‐specific).
- Extent of conceptualization (a specific conceptualization of self‐compassion or a broader, more generalized conceptualization of compassion).
- Text passages that refer to conceptualizations and their theoretical foundations (e.g., prior conceptualizations upon which the current conceptualization was based, theoretical or empirical foundations for including certain aspects).
- The authors of other conceptualizations.
- Information relevant for quality appraisal and the risk of bias assessment.

#### 3.3.4 Assessment of Risk of Bias

Several quality assessment procedures will be employed to identify the potential limitations of the included primary studies. Regarding the potential for reporting bias, it cannot be assumed that unsustainable conceptualizations have not been published. Furthermore, it is possible that authors may have omitted important findings from previous research when justifying their conceptualization. To counter this risk, we will evaluate the quality of eligible primary empirical studies by employing the Mixed‐Methods Appraisal Tool (MMAT; Hong et al. 2018). This tool was designed to assess both qualitative and quantitative studies and comprises seven questions for each study (two general questions and five relating to the specific study type). The three possible answers for each question are *Yes*, *No*, and *Can't tell*, with a column for comments. The MMAT will be used to assess the quality of studies in the following areas: the unambiguousness of the research questions and fit of the selected data, as well as the method for answering the research questions (for qualitative, quantitative, and mixed‐methods studies); the appropriateness of the sample and the instruments used, as well as the implementation of randomization or group formation (for quantitative studies); the completeness of the reported data (for quantitative studies); the appropriateness of the interpretation of the results and conclusions drawn from the data (for qualitative, quantitative, and mixed‐methods studies). For nonempirical studies, we developed a set of criteria concerning the authorship (transparency, affiliations), the content (are the arguments logical and supported by evidence, is this evidence correctly reported), and the publisher (specialized on scientific content or not) of the respective document. The response options have been adapted to the MMAT procedure. In addition, a statement about possible conflicts of interest or bias among the authors will be given for each included study (empirical and nonempirical).

To deal with possible publication bias, our systematic search covers a wide range of databases as suggested by MacDonald et al. (2024). Furthermore, we include gray literature in the search process and analyze the amount of conceptualizations reported in gray literature and in formally published work.

#### 3.3.5 Criteria for Determination of Independent Findings

The following measures will be employed to ensure the independence of data reported in eligible primary studies:

- Only those conceptualizations whose origins are clearly referenced by the authors will be included in the analyses. Therefore, eligible studies must clarify which of the presented conceptualizations were originally developed by the authors and which components were derived from other conceptualizations.
- If a single author produced more than one conceptualization (e.g., Neff 2003 and Neff 2016), each conceptualization will be included individually to facilitate the study of subsequent theoretical developments and account for components that may have been changed. However, the relations among interdependent conceptualizations will be presented transparently.

#### 3.3.6 Synthesis Procedures and Statistical Analysis

To provide a comprehensive overview of the findings of the systematic review for conceptualizations of general and domain‐specific self‐compassion, a descriptive evaluation of the following data is planned: publication year, document type, country of origin (records and authors), profession of authors, document focus, conceptualization area, and extent of the conceptualization.

Furthermore, an exploratory analysis of the conditional probability between the variables of document type and area of conceptualization will be conducted. Considering the preliminary literature search (nonsystematic, conducted in preparation for this study to ensure a comprehensive understanding of the issue), it seems that domain‐specific conceptualizations are more often found in empirical studies, while conceptualizations of general self‐compassion are more often found in solely qualitative theoretical studies.

Chi‐square tests will also be conducted to examine the interdependence of the document type and the area of conceptualization. If the chi‐square test is statistically significant, the strength of the relationship will be determined using Cramér's V. All analyses will be conducted using R (v4.3.2 or later; R Core Team 2024), presented as (cross) tables and visualized with appropriate chart forms (e.g., pie or bar charts).

##### 3.3.6.1 Methods to Achieve Review Objectives a and b

To answer the research questions about existing conceptualizations of general and domain‐specific self‐compassion, a comprehensive overview will be presented in tabular form, including information about the authors (country of origin and profession) and publication year, as well as a quote of the respective conceptualization.

##### 3.3.6.2 Methods to Achieve Objectives c and d

A systematic inductive approach will be employed to extract the following data:

- Components of both the general and domain‐specific conceptualizations of self‐compassion.
- Relationships and interactions among the components of general and domain‐specific self‐compassion.
- Theoretical or empirical foundations for the conceptualizations of general and domain‐specific self‐compassion.

The inductive category development procedure is a subtype of qualitative content analysis (Mayring 2022) used to systematically assess and categorize the content of a specific material. Before conducting qualitative content analysis, several parameters will be defined, including the *coding unit* (i.e., the object of analysis), the *context unit* (i.e., the context from which the object of analysis originates; in this case, the entire record), the *recording unit* (i.e., all sources to be evaluated within the framework of the analysis), the *selection criterion* (i.e., the selection of text fragments to be categorized), and the *level of abstraction* (i.e., the proximity of categories to the text and/or their abstraction level; Mayring and Fenzl 2019).

Starting with this analysis, the first text material will be processed, and initial categories will be formulated. The following material will then either be assigned to these or new categories. If the number of new categories decreases, the categories will be revised, and if necessary, the level of abstraction will be adjusted. The identified categories will be summarized into superordinate categories for interpretation (Mayring 2022).

The program QCAmap, which was developed for qualitative content analysis with teams of coders, will be used to facilitate the inductive category development process.

##### 3.3.6.3 Inductive Category Development Strategy to Achieve Objective c

Three phases of inductive category development will be conducted to identify the (1) components of general self‐compassion in previous conceptualizations, (2) the relationships and interactions among these components, and (3) theoretical foundations of these components.

The selection criteria and level of abstraction of the first inductive category development phase will be as follows:

- Coding unit: Text segments and illustrations taken from eligible primary studies that contain conceptualizations of general self‐compassion.
- Context unit: The full report.
- Recording unit: All eligible primary studies that contain conceptualizations of general self‐compassion.
- Definition of selection criterion: Components of conceptualizations that differ in quality, content, and process.
- Level of abstraction: Descriptions of psychological processes (including cognitive, emotional, perceptual, and mnemonic processes), behavioral processes, or prerequisites for self‐compassion.

After coding all eligible primary studies, the identified categories will be analyzed again to ascertain whether they can be consolidated into superordinate categories. The results of this analysis will be presented in tabular form, including the identified (superordinate) categories and examples. Information about the frequency of specific categories and intercoder agreement will be reported. This step will be conducted in the same way for all subsequent analyses and thus will also apply to the following inductive category development phases.

To identify the relationships and interactions among the components of general self‐compassion, an inductive category development phase will be conducted with the following boundary definitions:

- Coding unit: Text segments and illustrations taken from eligible primary studies that contain conceptualizations and in‐depth descriptions of the self‐compassion process.
- Context unit: The full report.
- Recording unit: All eligible primary studies that contain models of self‐compassion (i.e., that make assumptions about the relationships or interactions among distinct components of self‐compassion).
- Definition of selection criterion: Interactions among the individual components of conceptualizations.
- Level of abstraction: Descriptions of the interactions or interdependent processes that (are assumed to) occur among the individual components of self‐compassion.

The theoretical foundations of the conceptualizations will be categorized inductively with the following boundary definitions:

- Coding unit: Text segments and illustrations taken from eligible primary studies that contain information about the theoretical foundation of a conceptualization.
- Context unit: The full report.
- Recording unit: All eligible primary studies that contain conceptualizations of general self‐compassion.
- Definition of selection criterion: Theoretical foundations for the conceptualizations.
- Level of abstraction: Descriptions of psychological theories, empirical findings, or other critical remarks about earlier conceptualizations.

The inductive category development of components, the interactions among components, and the theoretical foundations of the conceptualizations of general self‐compassion will be followed by an analysis of the frequencies of identified categories. Components that occur frequently and are sufficiently theoretically sound will be combined into the comprehensive conceptualization of general self‐compassion. Assumptions about interactions among the selected components will be derived and integrated into the new comprehensive conceptualization.

One of the review authors (D.R.) will lead the process of integrating the components into a new conceptualization since they are experienced in the field of self‐compassion.

##### 3.3.6.4 Inductive Category Development Strategy to Achieve Objective d

To identify the components of the domain‐specific conceptualizations of self‐compassion, an inductive category development phase will be conducted with the following boundary definitions:

- Coding unit: Text segments and illustrations taken from eligible primary studies that contain domain‐specific conceptualizations of self‐compassion.
- Context unit: The full report.
- Recording unit: All eligible primary studies that contain domain‐specific conceptualizations of self‐compassion.
- Definition of selection criterion: Components of domain‐specific conceptualizations that differ in quality, content, and process.
- Level of abstraction: Descriptions of psychological processes (including cognitive, emotional, perceptual, and mnemonic processes), behavioral processes, or prerequisites of self‐compassion, including their integration into domain‐specific contexts (e.g., perception of mistakes at work, negative feedback about one's body, social conflicts, etc.) and domain‐specific processes or constructs (e.g., body image, leader identity, etc.).

The inductive category development phases will be followed by an analysis of the identified components of the domain‐specific conceptualizations. During this step, we will identify deviations from the general conceptualizations of self‐compassion among these categories to determine the nature of these domain‐specific components. The findings regarding the disparities between domain‐specific conceptualizations and the general conceptualization of the construct will be used as a foundation to identify relevant extensions for self‐compassion in educational and learning settings.

For developing a domain‐specific conceptualization of academic self‐compassion, concepts from the academic field that might be relevant for the self‐compassion process will be identified from established theories in academic research on learning, self‐monitoring, and self‐reactions in relation to academic performance. This process will be led by review authors F.P., C.D., and M.B., who have expertise in the field of learning and education. Furthermore, assumptions about the relationships among the academic conceptualizations and the general components of self‐compassion will be made, considering appropriate theoretical foundations.

The domain‐specific extensions and modifications of the general conceptualization of self‐compassion (including academic concepts and their relationships to general components of self‐compassion) will then be provided to a qualitative evaluation by experts from different professions (e.g., researchers with an expertise for self‐compassion and emotion regulation in educational settings, practitioners such as teachers and learning therapists). The involvement of nonscientific experts will be based on the recommendations for public involvement in review work (e.g., Boote et al. 2011). These experts will be identified through an online search of current studies on self‐compassion in the academic field, as well as based on thematically related contributions at scientific conferences in recent years. The entire research process (incl. identification and contact) will be carefully documented and included into the final report.

The evaluation process involves the implementation of a multifaceted approach, integrating the techniques of focus groups (Breen 2006; Krueger and Casey 2014) and rating conferences (Keller et al. 2012). Approximately three external experts per professional group are invited to join the project. Within each focus group, a brief thematic introduction is provided, followed by the presentation of the developed conceptualization of academic self‐compassion. Subsequently, the experts will be posed several key questions, designed to assess the conceptualization's appropriateness and quality. These questions will be addressed individually by the experts using a 5‐point Likert scale. The questions should include, for example, the appropriateness of the individual components of the conceptualization as well as their completeness. The responses of the individual experts will then be discussed in plenary. The purpose of the individual rating is to ensure that each expert's viewpoint is included. The objective of the group discussion is not to reach a consensus, but rather to discuss the diverse perspectives and arguments of individual experts. This approach aligns with the principles of a guided interview, integrating the strengths of both qualitative and quantitative approaches.

The focus group meetings are planned as online video conferences, recorded, and transcribed using specific software to ensure a detailed and complete evaluation of the entire discussion. The transcripts will be made accessible via OSF. The evaluation of the focus group discussions will adhere to the procedure recommended by Krueger and Casey (2014). In this procedure, similar statements are grouped into categories, and the frequency of these categories, as well as the distribution across different professional groups, will be documented. If clear disagreements on aspects of the conceptualization of academic self‐compassion emerge within or between focus groups, a subsequent qualitative evaluation of the frequency of these differing viewpoints will follow. In this regard, the identified critical aspects will be presented in an online survey to a larger group of experts from the aforementioned professions, who will be asked for a quantitative assessment using a self‐developed Likert‐scale questionnaire. The statements from experts are then subjected to critical discussion within the team of review authors and employed to refine the proposed conceptualization of academic self‐compassion.

## Author Contributions

- Content: R.S.‐H. and D.R. for self‐compassion, F.P., M.B., and C.D. for learning and education.
- Systematic review methods: S.K.S.
- Statistical expertise: S.K.S., D.R., F.P.
- Information retrieval: All authors and review research assistants.

To assess the potential impact of the researcher team on the outcomes of this systematic literature review, an exposition of the researchers' characteristics and reflexivity is provided below, as outlined by the SRQR guidelines (O'Brien et al. 2014). During the study's design phase, the authors engaged in continuous, critical dialog concerning the formulation of the research questions and planned methodological approaches. The present project is being carried out as part of the first author's dissertation (R.S.‐H.). The first author has received a solid scientific education that she is currently refining as part of her doctoral studies while having a non‐permanent position at the university. In addition to her academic pursuits, she has also accumulated extensive clinical experience during her training in cognitive behavioral therapy. S.K.S. is an assistant professor in the field of clinical psychology and head of an independent research group. She adds her expertise on systematic reviews and self‐compassion to this project. C.D. is currently pursuing her doctorate in the field of psychology, with a focus on self‐regulated learning. D.R. is the head of a research group (associate professor equivalent) in the field of psychological methods. She adds her expertise on systematic reviews and self‐compassion to this project. M.B. is a postdoctoral researcher whose area of expertise concerns metacognitive and motivational aspects of successful learning. F.P. serves as the principal professor of her research unit, with R.S.‐H. and C.D. being her doctoral students. F.P. has a high level of expertise in the field of learning and many years of experience in conducting qualitative and quantitative research projects. M.B. acts as an associate researcher at F.P.'s chair. All authors are female and from Germany. The project is not sponsored or funded by any third party.

## Conflicts of Interest

The authors declare no conflicts of interest.

## Preliminary Timeframe

Searches for eligible studies will start immediately after the approval of the protocol, searches will be performed on a single day in all databases. If the review takes more than 12 months, a search update is planned to ensure that all relevant studies published after the initial search are included (MacDonald et al. 2024).

- Screening the results from the literature search: 3 to 6 months.
- Extraction of data from eligible primary studies: 2 to 4 months.
- Analysis: 2 to 4 months.
- Preparation of final review report: 3 months.

## Sources of Support

### Internal Sources

No sources of support provided.

### External Sources

No sources of support provided.

## Supporting information

Appendix.
