# Supplementary material for: PROTOCOL: Summarizing and Critically Evaluating the Concepts of Self‐Compassion: A Systematic Review of Conceptualizations
Source: Campbell Syst Rev. 2025 Jul 13;21(3):e70054. doi: 10.1002/cl2.70054 (PMC12255902; doi:10.1002/cl2.70054)
Supplement: Supplementary file 1 — Appendix. [file CL2-21-e70054-s001.docx]

# Appendices

**Appendix 1**. Search Strategies.

Web of Science databases

- Web of Science Core Collection

| Set# | Search syntax |
| --- | --- |
| S1 | ((TI=((self-compassion OR “self compassion” OR selfcompassion OR self*compassion OR self?compassion) AND (Defin* OR Concept* OR theor* OR model* OR expla* OR characteriz* OR clarif* OR determin* OR formali* OR terminolog* OR elucidat* OR outlin* OR construct* OR descri* OR understand* OR breakdown OR “breaking down” OR “broken down” OR account* OR establish* OR analyz* OR dissect* OR approach* OR propos* OR discuss* OR consider* OR idea* OR term*) )) OR AB=((self-compassion OR “self compassion” OR selfcompassion OR self*compassion OR self?compassion) AND (Defin* OR Concept* OR theor* OR model* OR expla* OR characteriz* OR clarif* OR determin* OR formali* OR terminolog* OR elucidat* OR outlin* OR construct* OR descri* OR understand* OR breakdown OR “breaking down” OR “broken down” OR account* OR establish* OR analyz* OR dissect* OR approach* OR propos* OR discuss* OR consider* OR idea* OR term*) )) OR AK=((self-compassion OR “self compassion” OR selfcompassion OR self*compassion OR self?compassion) AND (Defin* OR Concept* OR theor* OR model* OR expla* OR characteriz* OR clarif* OR determin* OR formali* OR terminolog* OR elucidat* OR outlin* OR construct* OR descri* OR understand* OR breakdown OR “breaking down” OR “broken down” OR account* OR establish* OR analyz* OR dissect* OR approach* OR propos* OR discuss* OR consider* OR idea* OR term*) ) Timespan: 2003-01-01 to 2024-05-01 (Publication Date) |

PubPsych databases

- PSYNDEX
- NARCIS
- ISOC-Psicologia
- Pascal
- PsychOpen

| Set# | Search syntax |
| --- | --- |
| S1 | TI:(self-compassion OR “self compassion” OR selfcompassion OR self*compassion OR self?compassion) AND (Defin* OR Concept* OR theor* OR model* OR expla* OR characteriz* OR clarif* OR determin* OR formali* OR terminolog* OR elucidat* OR outlin* OR construct* OR descri* OR understand* OR breakdown OR “breaking down” OR “broken down” OR account* OR establish* OR analyz* OR dissect* OR approach* OR propos* OR discuss* OR consider* OR idea* OR term*) PY>=2003 |
| S2 | AB:(self-compassion OR “self compassion” OR selfcompassion OR self*compassion OR self?compassion) AND (Defin* OR Concept* OR theor* OR model* OR expla* OR characteriz* OR clarif* OR determin* OR formali* OR terminolog* OR elucidat* OR outlin* OR construct* OR descri* OR understand* OR breakdown OR “breaking down” OR “broken down” OR account* OR establish* OR analyz* OR dissect* OR approach* OR propos* OR discuss* OR consider* OR idea* OR term*) PY>=2003 |
| S3 | SW:(self-compassion OR “self compassion” OR selfcompassion OR self*compassion OR self?compassion) AND (Defin* OR Concept* OR theor* OR model* OR expla* OR characteriz* OR clarif* OR determin* OR formali* OR terminolog* OR elucidat* OR outlin* OR construct* OR descri* OR understand* OR breakdown OR “breaking down” OR “broken down” OR account* OR establish* OR analyz* OR dissect* OR approach* OR propos* OR discuss* OR consider* OR idea* OR term*) PY>=2003 |

EBSCOhost databases

- APA PsycArticles
- APA PsycInfo
- OpenDissertations

| Set# | Search syntax |
| --- | --- |
| S1 | TI ( (self-compassion OR “self compassion” OR selfcompassion OR self*compassion OR self?compassion) AND (Defin* OR Concept* OR theor* OR model* OR expla* OR characteriz* OR clarif* OR determin* OR formali* OR terminolog* OR elucidat* OR outlin* OR construct* OR descri* OR understand* OR breakdown OR “breaking down” OR “broken down” OR account* OR establish* OR analyz* OR dissect* OR approach* OR propos* OR discuss* OR consider* OR idea* OR term*) ) OR AB ( (self-compassion OR “self compassion” OR selfcompassion OR self*compassion OR self?compassion) AND (Defin* OR Concept* OR theor* OR model* OR expla* OR characteriz* OR clarif* OR determin* OR formali* OR terminolog* OR elucidat* OR outlin* OR construct* OR descri* OR understand* OR breakdown OR “breaking down” OR “broken down” OR account* OR establish* OR analyz* OR dissect* OR approach* OR propos* OR discuss* OR consider* OR idea* OR term*) ) OR KW ( (self-compassion OR “self compassion” OR selfcompassion OR self*compassion OR self?compassion) AND (Defin* OR Concept* OR theor* OR model* OR expla* OR characteriz* OR clarif* OR determin* OR formali* OR terminolog* OR elucidat* OR outlin* OR construct* OR descri* OR understand* OR breakdown OR “breaking down” OR “broken down” OR account* OR establish* OR analyz* OR dissect* OR approach* OR propos* OR discuss* OR consider* OR idea* OR term*) ) from 2003 to 2024 |

- ERIC

| Set# | Search syntax |
| --- | --- |
| S1 | TI ( (self-compassion OR “self compassion” OR selfcompassion OR self*compassion OR self?compassion) AND (Defin* OR Concept* OR theor* OR model* OR expla* OR characteriz* OR clarif* OR determin* OR formali* OR terminolog* OR elucidat* OR outlin* OR construct* OR descri* OR understand* OR breakdown OR “breaking down” OR “broken down” OR account* OR establish* OR analyz* OR dissect* OR approach* OR propos* OR discuss* OR consider* OR idea* OR term*) ) OR AB ( (self-compassion OR “self compassion” OR selfcompassion OR self*compassion OR self?compassion) AND (Defin* OR Concept* OR theor* OR model* OR expla* OR characteriz* OR clarif* OR determin* OR formali* OR terminolog* OR elucidat* OR outlin* OR construct* OR descri* OR understand* OR breakdown OR “breaking down” OR “broken down” OR account* OR establish* OR analyz* OR dissect* OR approach* OR propos* OR discuss* OR consider* OR idea* OR term*) ) OR DE ( (self-compassion OR “self compassion” OR selfcompassion OR self*compassion OR self?compassion) AND (Defin* OR Concept* OR theor* OR model* OR expla* OR characteriz* OR clarif* OR determin* OR formali* OR terminolog* OR elucidat* OR outlin* OR construct* OR descri* OR understand* OR breakdown OR “breaking down” OR “broken down” OR account* OR establish* OR analyz* OR dissect* OR approach* OR propos* OR discuss* OR consider* OR idea* OR term*) ) from 2003 to 2024 |

PubMed databases

- Medline

| Set# | Search syntax |
| --- | --- |
| S1 | (self-compassion[Title/Abstract] OR "self compassion"[Title/Abstract] OR selfcompassion[Title/Abstract] OR self*compassion[Title/Abstract] OR self?compassion[Title/Abstract]) AND (Defin*[Title/Abstract] OR Concept*[Title/Abstract] OR theor*[Title/Abstract] OR model*[Title/Abstract] OR expla*[Title/Abstract] OR characteriz*[Title/Abstract] OR clarif*[Title/Abstract] OR determin*[Title/Abstract] OR formali*[Title/Abstract] OR terminolog*[Title/Abstract] OR elucidat*[Title/Abstract] OR outlin*[Title/Abstract] OR construct*[Title/Abstract] OR descri*[Title/Abstract] OR understand*[Title/Abstract] OR breakdown[Title/Abstract] OR "breaking down"[Title/Abstract] OR "broken down"[Title/Abstract] OR account*[Title/Abstract] OR establish*[Title/Abstract] OR analyz*[Title/Abstract] OR dissect*[Title/Abstract] OR approach*[Title/Abstract] OR propos*[Title/Abstract] OR discuss*[Title/Abstract] OR consider*[Title/Abstract] OR idea*[Title/Abstract] OR term*[Title/Abstract])  No restrictions regarding the publication period possible |

SAGE Journals

| Set# | Search syntax |
| --- | --- |
| S1 | “(self-compassion OR “self compassion” OR selfcompassion OR self*compassion OR self?compassion) AND (Defin* OR Concept* OR theor* OR model* OR expla* OR characteriz* OR clarif* OR determin* OR formali* OR terminolog* OR elucidat* OR outlin* OR construct* OR descri* OR understand* OR breakdown OR “breaking down” OR “broken down” OR account* OR establish* OR analyz* OR dissect* OR approach* OR propos* OR discuss* OR consider* OR idea* OR term*)” in Title from January 2003 to May 2024 |
| S2 | “(self-compassion OR “self compassion” OR selfcompassion OR self*compassion OR self?compassion) AND (Defin* OR Concept* OR theor* OR model* OR expla* OR characteriz* OR clarif* OR determin* OR formali* OR terminolog* OR elucidat* OR outlin* OR construct* OR descri* OR understand* OR breakdown OR “breaking down” OR “broken down” OR account* OR establish* OR analyz* OR dissect* OR approach* OR propos* OR discuss* OR consider* OR idea* OR term*)” in Abstract from January 2003 to May 2024 |
| S3 | “(self-compassion OR “self compassion” OR selfcompassion OR self*compassion OR self?compassion) AND (Defin* OR Concept* OR theor* OR model* OR expla* OR characteriz* OR clarif* OR determin* OR formali* OR terminolog* OR elucidat* OR outlin* OR construct* OR descri* OR understand* OR breakdown OR “breaking down” OR “broken down” OR account* OR establish* OR analyz* OR dissect* OR approach* OR propos* OR discuss* OR consider* OR idea* OR term*)” in Keywords from January 2003 to May 2024 |

Scopus

| Set# | Search syntax |
| --- | --- |
| S1 | PUBYEAR AFT 2003 (self-compassion OR self.compassion OR selfcompassion OR self*compassion) AND (Defin* OR Concept* OR theor* OR model* OR expla* OR characteriz* OR clarif* OR determin* OR formali* OR terminolog* OR elucidat* OR outlin* OR construct* OR descri* OR understand* OR breakdown OR breaking.down OR broken.down OR account* OR establish* OR analyz* OR dissect* OR approach* OR propos* OR discuss* OR consider* OR idea* OR term*) |

**Appendix 2.** Screening guidelines.

***Resolving jingle-jangle fallacies in self-compassion research***
Screening guidance at title/abstract level (version 1.0 – 2024/09/14)

| \| **Population** \| \| --- \| \| **Include:**   - all populations/samples regardless of age, gender, other socio-demographic characteristics, mental or physical health status \|  \| - Publication date limited to 2003 to present - No restriction regarding language of abstract (please use DeepL for Non-English or Non-German abstracts) - Exclude any Non-English or Non-German documents  **🡪 This is relevant at full text level.** \| \| --- \| | \| **Study Design** \| \| --- \| \| **Include:**   - *empirical research*: cross-sectional or longitudinal designs using quantitative and qualitative methods, experimental studies including randomized controlled trials (RCTs) and cluster-randomized trials (cRCTs), pilot studies, correlational studies, descriptive studies - *theoretical research*: narrative reviews, position papers, discussions - systematic reviews and meta-analyses - theses, doctoral dissertations - research reports, preprints, preregistrations, protocols - commentaries - conference papers and posters - Letters to the editor, editorials - Book chapters \| |
| --- | --- | --- | --- | --- | --- | --- |

| **Outcomes** |
| --- |
| **Include:**   - a **conceptualization** of general or domain-specific self-compassion is **presented/announced to be** **presented** which is either   - empirically tested ***OR***   - merely based on theoretical considerations   e.g.: “. In the process, we offer a dynamic, process-based conceptualization of self-compassion….” “This article defines and examines the construct of self-compassion.”   - it is announced that general or domain-specific self-compassion (its nature, constitution...) or conceptualizations **will be discussed** in the full text record e.g.: “This meta-narrative review, conducted according to the RAMESES standards, critically examines the construct of self-compassion to determine if it is an accurate target variable to mitigate work-related stress and promote compassionate caregiving in healthcare providers.” “We examine self-compassion in terms of pragmatic analysis and process-based theory that brings together different self-compassion approaches.” - the **conceptual ambiguity** of the construct of self-compassion is discussed e.g.: “The last few decades have seen an explosion of self-compassion research, and yet the measurement of self-compassion remains fiercely debated.” “Even though compassion for others and for the self are important indicators of mental and physical health and well-being, scientists vary greatly in defining them.” “Yet, there is lack of consensus on definition and a paucity of psychometrically robust measures of this construct.” - conceptualizations can encompass   - descriptions of the components of general or domain-specific self-compassion e.g.: “Self-compassion, therefore, involves being touched by and open to one’s own suffering, not avoiding or disconnecting from it, generating the desire to alleviate one’s suffering and to heal oneself with kindness. Self-compassion also involves offering nonjudgmental understanding to one’s pain, inadequacies and failures, so that one’s experience is seen as part of the larger human experience.”   - representations of theoretically derived relationships between different components of general or domain-specific self-compassion e.g.: “…self-compassion be conceptualized as a dynamic process that begins with mindful awareness of personal suffering (noticing), followed by appreciating and empathizing with one's own pain (feeling) which culminates in a response to alleviate it (acting).”   **Exclude:**   - studies reporting on non-scientific/vernacular conceptualizations of self-compassion (based on input from the general population or specific populations)   e.g.: “…this study aims to explore the meaning of self-compassion experienced by patients with multiple sclerosis. […] patients with Multiple sclerosis were selected purposefully and interviewed individually. Qualitative content analysis was used for data analysis […]. These categories express the characteristics and meaning of self-compassion in patients with multiple sclerosis. […] Results of the present study showed that new dimensions of self-compassion were found by exploring multiple sclerosis patients' experiences, which added to the suggested dimensions of others.”   - studies only presenting theories on the development of self-compassion in mankind or within a person   **🡪 This distinction cannot be accurately assessed at T/A-level yet and is to be assessed at full text level.**   - studies only presenting models that represent the relationship between general or domain-specific self-compassion and other constructs (e.g., moderation or mediation models, psychological network models) |

| **T/A Screening guidance** | Labelling **at full text level** (no T/A)- unclear/”special”   - Preregistrations 🡪 label “preregistration”, protocols 🡪 label “protocol“, research reports 🡪 label “report”, preprints 🡪 label “preprint”, dissertations 🡪 label „dissertation“, theses 🡪 label “theses”, conference paper/poster 🡪 label “conference contribution”, book chapter 🡪 label “chapter”, Commentaries 🡪 label “commentary”, editorials 🡪 label “editorial”, letters to the editor 🡪 label “letter” - if full text is not retrievable 🡪 label “full text missing” and put the reference in the following file: [Missing Abstracts/Full-texts](https://docs.google.com/document/d/1aL4dq4XDuGOLio8X5E9XZD_6U2bQnH9HRyBKeB4aDP0/edit?usp=sharing) - if full text is in another language than German or English 🡪 label “other language”   Exclusion reasons at full text level (not T/A): pleas check in the following order   1. no own conceptualization of general or domain-specific self-compassion is presented  🡪 label with exclusion reason in Rayyan: “No own conceptualization”. 2. no preexisting definition or model of general or domain-specific self-compassion is further developed/modified  🡪 label with exclusion reason in Rayyan: “No own conceptualization”. 3. the origin of the presented conceptualization(s) is not clearly stated (by which author/if it’s an own conceptualization/if it’s an adaptation or extension of existing work) 🡪 label with exclusion reason in Rayyan: “No own conceptualization” 4. the study is only presenting a theory and not a model or definition of general or domain-specific self-compassion: Theories are concerned with the development of general or domain-specific self-compassion and/or its embedding in broader frameworks of emotion regulation, mental health etc.  e.g.: “Social Mentality Theory (SMT; Gilbert 2005) posits that self-compassion is a state of mind that emerges from mammalian biosocial roles involving caregiving and care-seeking, while self-criticism emerges from evolved social roles that protect us from social threats.” 🡪 label with exclusion reason in Rayyan: “Theory”   🡪 Please use the following file to document references that are used as sources of conceptualizations presented in primary studies (in case no own conceptualization) is presented: [Full-texts_Only references](https://docs.google.com/spreadsheets/d/12jQT6ojhm5dYq4iNnEVGVRMvAzbGitzD2uQOJHgE6GE/edit?usp=sharing) |
| --- | --- |
| - Conducted in Rayyan - Important: Use labels in Rayyan as defined in this guidance (please copy and paste them to avoid small mismatches hindering label fusion between raters) - Team setting:   - References are not allocated and can be screened by any team member   - Please select ‘at most 1’ for ‘maximum collaborator decisions’ to see reports that need screening   Labelling (at T/A level) – unclear/”special”   - Unclear studies 🡪 save for yourself as ‘maybe’ and review again/discuss with other raters, then make a final decision for inclusion/exclusion  We will have regular meetings to discuss questions. - Same study appearing multiple times 🡪 label “Duplicate” - Correctionals/Erratums 🡪 exclude and add a comment to the original study “Correction was published: DOI:…………….” - Studies with no abstract 🡪 search for abstract using Google or Pubmed & put it in a comment in Rayyan, if you cannot find an abstract 🡪 label “no abstract” and put the reference in the following file: [Missing Abstracts/Full-texts](https://docs.google.com/document/d/1aL4dq4XDuGOLio8X5E9XZD_6U2bQnH9HRyBKeB4aDP0/edit?usp=sharing) |  |

**Appendix 3.** Coding Scheme**.**

| **Coding Scheme** | | | |
| --- | --- | --- | --- |
| *Metadata* | | | |
| Document complete citation: | |  |  |
| DOI: |  | |  |
|  | | | |
| *General Information* | | | |
| Document Type (Tick as applicable, multiple answers possible):   \| Empirical research (1) \| Cross-Sectional Design (1)  Longitudinal design (2)  Quantitative Data (3)  Qualitative Data (4)  Experimental Study (randomized controlled trial or cluster-randomized trial) (5)  Correlational study (6)  Descriptive study (7)  Moderation/Mediation Analysis (8)  Structural equation modelling (SEM) (9)  Instrument validation (10) \| \| --- \| --- \| \| Theoretical research (2) \| Commentary (1)  Position paper (2)  Discussion (3) \| \| Meta study (3) \| Qualitative Review (including narrative reviews, mapping reviews, scoping reviews, systematic literature reviews) (1)  Qualitative Review (meta-analysis) (2)  Mixed Studies/Methods Review (3)  Umbrella review (4) \| \| Grey literature (4) \| Thesis (1)  Doctoral dissertation (2)  Conference paper (3)  Conference poster (4) \| \| Other (5) \| Commentary (1)  Letter to the editor (2)  Editorial (3)  Book Chapter (4)  Research Report (5)  Preprint (6)  Preregistration (7)  Study Protocol (8) \| | | | |
| Language   \| English (1) \| German (2) \| \| --- \| --- \| | | | |
| \| Record \|  \| \| \| --- \| --- \| --- \| \| Authors \| Author 1: \|  \| \| Author 2: \|  \| \| Author 3: \|  \| \| … \|  \|   Country of Origin | | | |
| \| Author 1: \|  \| \| --- \| --- \| \| Author 2: \|  \| \| Author 3: \|  \| \| … \|  \|   Profession(s) of authors (e.g., researcher, practitioner, educator, politician…) | | | |
| Primary focus of the Article   \| Self-compassion (1) \| \| \| --- \| --- \| \| Other (2): \|  \| | | | |
| Area of original Conceptualization   \| General self-compassion (1) \| \|  \| \| --- \| --- \| --- \| \| Domain-specific self-compassion (2) \| \| Please note the domain: \| \| Both (3) \|  \| \| | | | |
| If further non-original conceptualizations are included, state their references   \| No. \| Reference \| \| --- \| --- \| \|  \|  \| | | | |
| *Information on general conceptualization*  (only refers to the original conceptualization by authors) | | | |
| Extent of Conceptualization  Self-compassion (1)  General compassion including compassion towards the self (2) | | | |
| Direct citation of Conceptualization  (including page number – also add figures) | | | |
| Processes between components  Should any assumptions be made regarding the interaction of the individual components or further descriptions of the process of self-compassion, please note this here (including page number, also add figures): | | | |
| Theoretical foundations   \| Presented (1) \| Please note any presented theoretical foundations (including page number) \| \| --- \| --- \| \| Non presented (2) \| \| | | | |
|  | | | |
| *Information on domain-specific conceptualization*  (only refers to the original conceptualization by authors) | | | |
| Direct citation of Conceptualization  (including page number – also add figures) | | | |
| Processes between components  Should any assumptions be made regarding the interaction of the individual components or further descriptions of the process of self-compassion, please note this here (including page number, also add figures): | | | |
| Theoretical foundations   \| Presented (1) \| Please note any presented theoretical foundations (including page number) \| \| --- \| --- \| \| Non presented (2) \| \| | | | |

| *Quality assessment* |
| --- |
| *Empirical Studies* |
| Mixed Methods Appraisal Tool (MMAT), version 2018 |
| \| **Category of study designs** \| **Methodological quality criteria** \| **Responses** \| \| \| \| \| --- \| --- \| --- \| --- \| --- \| --- \| \| Yes \| No \| Can’t tell \| Comments \| \| Screening questions  (for all types) \| S1. Are there clear research questions? \|  \|  \|  \|  \| \| S2. Do the collected data allow to address the research questions? \|  \|  \|  \|  \| \| *Further appraisal may not be feasible or appropriate when the answer is ‘No’ or ‘Can’t tell’ to one or both screening questions.* \| \| \| \| \| \| Qualitative \| 1.1. Is the qualitative approach appropriate to answer the research question? \|  \|  \|  \|  \| \| 1.2. Are the qualitative data collection methods adequate to address the research question? \|  \|  \|  \|  \| \| 1.3. Are the findings adequately derived from the data? \|  \|  \|  \|  \| \| 1.4. Is the interpretation of results sufficiently substantiated by data? \|  \|  \|  \|  \| \| 1.5. Is there coherence between qualitative data sources, collection, analysis and interpretation? \|  \|  \|  \|  \| \| Quantitative randomized controlled trials \| 2.1. Is randomization appropriately performed? \|  \|  \|  \|  \| \| 2.2. Are the groups comparable at baseline? \|  \|  \|  \|  \| \| 2.3. Are there complete outcome data? \|  \|  \|  \|  \| \| 2.4. Are outcome assessors blinded to the intervention provided? \|  \|  \|  \|  \| \| 2.5 Did the participants adhere to the assigned intervention? \|  \|  \|  \|  \| \| Quantitative non-randomized \| 3.1. Are the participants representative of the target population? \|  \|  \|  \|  \| \| 3.2. Are measurements appropriate regarding both the outcome and intervention (or exposure)? \|  \|  \|  \|  \| \| 3.3. Are there complete outcome data? \|  \|  \|  \|  \| \| 3.4. Are the confounders accounted for in the design and analysis? \|  \|  \|  \|  \| \| 3.5. During the study period, is the intervention administered (or exposure occurred) as intended? \|  \|  \|  \|  \| \| Quantitative descriptive \| 4.1. Is the sampling strategy relevant to address the research question? \|  \|  \|  \|  \| \| 4.2. Is the sample representative of the target population? \|  \|  \|  \|  \| \| 4.3. Are the measurements appropriate? \|  \|  \|  \|  \| \| 4.4. Is the risk of nonresponse bias low? \|  \|  \|  \|  \| \| 4.5. Is the statistical analysis appropriate to answer the research question? \|  \|  \|  \|  \| \| Mixed methods \| 5.1. Is there an adequate rationale for using a mixed methods design to address the research question? \|  \|  \|  \|  \| \| 5.2. Are the different components of the study effectively integrated to answer the research question? \|  \|  \|  \|  \| \| 5.3. Are the outputs of the integration of qualitative and quantitative components adequately interpreted? \|  \|  \|  \|  \| \| 5.4. Are divergences and inconsistencies between quantitative and qualitative results adequately addressed? \|  \|  \|  \|  \| \| 5.5. Do the different components of the study adhere to the quality criteria of each tradition of the methods involved? \|  \|  \|  \|  \| \| Hong QN, Pluye P, Fàbregues S, Bartlett G, Boardman F, Cargo M, Dagenais P, Gagnon M-P, Griffiths F, Nicolau B, O’Cathain A, Rousseau M-C, Vedel I. Mixed Methods Appraisal Tool (MMAT), version 2018. Registration of Copyright (#1148552), Canadian Intellectual Property Office, Industry Canada. \| \| \| \| \| \| |
| Do the authors state a disclosure of possibilities of any conflict of interest that may exist?  Yes  No  If yes, which possible COI’s are stated by the authors that might be relevant to their conceptualization of (domain-specific) self-compassion? |
| *Non-empirical documents (e.g., position papers, discussions, commentaries, editorials,…)* |
| Authorship   \| Are the authors of the document clearly stated with their full names? \| Yes  No  Can’t tell  Comments: ______________________ \| \| --- \| --- \| \| Is it stated that they're affiliated with a scientific institution? \| Yes  No  Can’t tell  Comments: ______________________ \| \| Is any other affiliation indicated (e.g. to a company or a political party?) \| Yes - Please state which kind of affiliation here: ______________________  No  Can’t tell  Comments: ______________________ \| |
| Content   \| Are the author's line of reasoning logically comprehensible? (Do the statements logically support the author’s conclusion?) \| Yes  No  Can’t tell  Comments: ______________________ \| \| --- \| --- \| \| Does the author also shed light on opposing views or limitations of his own argumentation? \| Yes  No  Can’t tell  Comments: ______________________ \| \| Do the author(s) support their statements with references that are presented in a way that is traceable for the reader (Name of author(s), publication year, title or DOI/other identification code)? \| Yes  No  Can’t tell  Comments: ______________________ \| |
| Publisher  Is the publisher of the document specialized in scientific literature?  Yes  No  Can’t tell  Comments: ______________________ |
| Do the authors state a disclosure of possibilities of any conflict of interest that may exist?  Yes  No  If yes, which possible COI’s are stated by the authors that might be relevant to their conceptualization of (domain-specific) self-compassion? |
